# Efficacy of Rapid Salivary C-Reactive Protein Test to Assess Early Changes in Malignancy in the Oral Cavity and Its Utility in Screening for Oral Cancer

**DOI:** 10.3390/dj13010034

**Published:** 2025-01-15

**Authors:** Vathsala Patil, Ravindranath Vineetha, Komal Smriti, Kalyana Chakravarthy Pentapati, Srikanth Gadicherla, Carnelio Sunitha

**Affiliations:** 1 Department of Oral Medicine & Radiology, Manipal College of Dental Sciences, Manipal Academy of Higher Education, Udupi 576104, Karnataka, India; vathsala.mcods@manipal.edu (V.P.); vineetha.manu@manipal.edu (R.V.); 2 Department of Public Health Dentistry, Manipal College of Dental Sciences, Manipal Academy of Higher Education, Udupi 576104, Karnataka, India; kalyan.cp@manipal.edu; 3 Department of Oral and Maxillofacial Surgery, Manipal College of Dental Sciences, Manipal Academy of Higher Education, Udupi 576104, Karnataka, India; srikanth.mds@manipal.edu; 4 Department of Oral Pathology and Microbiology, Manipal College of Dental Sciences, Manipal Academy of Higher Education, Udupi 576104, Karnataka, India; sunitha.carnelio@manipal.edu

**Keywords:** oral cancer, C-reactive protein, oral potentially malignant disorder, biomarker, saliva

## Abstract

**Background/Objectives:** The present study aimed to test the efficacy of the chair-side rapid salivary C-reactive protein assay kit in differentiating oral potentially malignant disorders (OPMDs) and oral cancer from normal mucosa using whole salivary samples. **Methods:** In this study, unstimulated saliva samples of cases (OPMDs and oral cancer) and controls (systemically healthy subjects) were used to detect CRP levels using a novel colorimetric, quantitative rapid assay kit. Kruskal–Wallis ANOVA with a post hoc Dunn’s test were applied to determine the difference in the mean and SD values between the case and control groups. ROC analysis was performed to identify the positive and negative likelihood ratios. **Results:** The mean calculated salivary CRP level in the oral cancer group was 4.21 ng/mL, in the OPMD group it was 2.51 ng/mL and in the healthy controls it was 0.7 ng/mL. Post hoc tests showed that the salivary CRP levels were significantly higher in the oral cancer and OPMD groups than in the controls. **Conclusions:** The present study showed that the novel rapid salivary test kit could significantly differentiate between the salivary CRP values of cases and controls but there was no significant difference between the OPMD and malignancy groups. It also showed higher sensitivity values, confirming the efficacy of this kit as a screening tool.

## 1. Introduction

Oral cancer is the sixth most common cancer with the highest rate of mortality and morbidity. Despite various emerging treatment modalities, the mortality rate has not been reduced, and the prognosis remains poor. The key reason for the high mortality rate remains the lack of early screening, resulting in more than two-thirds of patients being diagnosed at later stages of cancer [1]. A majority of oral cancers are preceded by certain early changes, known as oral potentially malignant disorders (OPMDs). They usually present with white patches, red patches and ulcerations in the oral mucosa. The early diagnosis and management of OPMDs are known to prevent cancer development in 88% of cases and enable downstaging of the disease and mortality rates [2,3,4]. Therefore, there is a need for cost-effective and efficient markers that can identify these lesions at an early stage. Previous studies have reported an association between cancer and inflammation. Chronic inflammation is known to play a crucial role in cancer initiation, promotion and progression, thereby increasing its overall incidence [5]. Recently, genomics and advances in the field of proteomics have facilitated the identification of various biomarkers in body fluids, like serum, saliva, cerebrospinal fluid and urine, for the diagnosis of several diseases like malignancies, auto immune conditions, Parkinsonism, etc. [6].

C-reactive protein (CRP) is a key marker for inflammation and is routinely evaluated in the serum during clinical practice. It was first described by Tillett and Francis in 1930. CRP plays multiple important roles in innate immunity and host defense. It binds to foreign bodies and damaged cells and triggers a complement cascade [7]. Serum CRP level is clinically used to assess inflammation and chronic infections in the body as its value rises after initial stimuli from infections and maintains a predictable level [8]. It can also be used as a non-invasive screening tool for malignant changes [5]. While inflammatory mediators such as interleukins show a lesser association between serum and salivary levels, the salivary level of CRP is known to mirror the serum CRP levels [9]. Evidence suggests that mean CRP levels are higher in patients with OPMDs and oral cancer at advanced tumor stages. Previous studies on evaluating salivary CRP values have utilized laboratory techniques that are time-consuming and cumbersome and deliver delayed results after the patients have left the referral centers [5,10]. Although these techniques are becoming popular and being adapted to clinical set-ups, their analysis is time-consuming and requires big set-ups. Salivary sample analysis is non-invasive, convenient and feasible for repeat sampling; hence, it is more favored compared to serum analysis, which is invasive, requires penetration of the skin and is associated with pain and discomfort. Salivary diagnostics are now being integrated to develop point-of-care devices and rapid test kits for diagnosis, clinical monitoring and treatment planning. Rapid analysis using modern biosensor technology like paper-based microfluidics have many benefits as they are user friendly, rapid, portable and reliable [11,12]. This study aimed to evaluate the efficacy of the chair-side colorimetric, quantitative rapid salivary C-reactive protein assay kit (Spotsense- Spot Healthcare Pvt. Ltd, Jharkhand, India) in differentiating oral potentially malignant disorders and oral cancer from normal mucosa using whole salivary samples. Ours is the first study wherein a chair-side novel CRP colorimetric kit is used in cases of oral potentially malignant disorders and oral malignancy.

## 2. Methodology

This study was conducted at the Department of Oral Medicine and Radiology, Manipal College of Dental Sciences, Manipal, in collaboration with Spot Healthcare Pvt. Ltd. The study was designed as a cross-sectional diagnostic accuracy test conducted from May 2023 to December 2023 following the STARD guidelines. The study was conducted in accordance with the tenets of the Helsinki Declaration (revised in 2013) and was approved by the Institutional Ethics Committee of Kasturba Medical College and Kasturba Hospital (IEC No:351/2022). This study was also registered in the clinical trial registry in India (Registry no. CTRI/2023/05/067894).

### 2.1. Sample Size Calculation

Sample size was estimated with G*power software (version 3). Considering an effect size of 0.4 (large effect size), which was based on the results of the preliminary pilot study on 20 samples for feasibility, and a power of 90% and 95% confidence interval for the three study groups, the sample size was estimated to be a minimum of 84 (n = 28 per group). However, an oversampling of 20% was used as the inclusion criteria included a positive histopathology report, which would be obtained at a later date.

### 2.2. Inclusion and Exclusion Criteria

All individuals visiting the Department of Oral Medicine and Radiology at Manipal College of Dental Sciences were screened for inclusion and exclusion criteria.

#### 2.2.1. Inclusion Criteria

Inclusion criteria for cases group

Patients with OPMDs and oral cancer were considered for the study.

Inclusion criteria for OPMD group

- Clinically diagnosed cases of leukoplakia/non-homogeneous leukoplakia–erythroplakia with histopathological diagnosis of hyperkeratosis/parakeratosis with or without dysplasia, with acanthosis, atrophy or inflammation.
- Clinically diagnosed cases of OSMF.
- Clinically and histopathologically proven cases of erosive variant of oral lichen planus.

Inclusion criteria for oral cancer group

The inclusion criteria for the oral cancer group were as follows:

- Histopathologically diagnosed cases of oral squamous cell carcinoma;
- Histopathologically proven cases of carcinoma in situ or invasive carcinoma.

Inclusion criteria for control group

The control group samples were selected from patients visiting the outpatient department. The criteria were as follows:

- Patients with no oral habits such as tobacco chewing, smoking, or alcohol consumption;
- Patients in good systemic health, not taking any medication for systemic illnesses;
- Patients who demonstrated good oral hygiene and were free from any oral infection or inflammation such as oral ulcers or inflamed gums.

#### 2.2.2. Exclusion Criteria for Case and Control Groups

Several exclusion criteria for the case and control groups were applied, as follows:

- Patients with underlying systemic diseases were excluded from the study;
- Patients taking medications that could alter CRP levels, pregnant and lactating women, mentally and physically challenged individuals, and those undergoing radiation or chemotherapy were also excluded;
- Patients with ulcers and inflammation in the oral cavity were not included in the study.

Detailed information regarding patient demographics and oral condition was collected using a printed data collection form. This process was overseen by two investigators, KS and VP, ensuring comprehensive data capture and accuracy in demographic and oral health information. Samples for salivary C-reactive protein (CRP) analysis were collected using a standardized protocol to ensure accuracy and reliability. The collection and analysis of salivary samples using the kit were conducted in a blinded manner, ensuring that the principal investigators (KS and VP) were unaware of the details. Participants were instructed to sit comfortably in an upright position, and those with dentures or removable prostheses removed them before collection to prevent any interference or contamination.

Salivary samples were collected around 10 AM using the spitting method. Approximately 2 to 3 mL of unstimulated saliva was gathered in Eppendorf vials. The saliva samples collected were used immediately without any storing, pre-processing or centrifuging. The analysis of salivary CRP levels was performed using a colorimetric, quantitative rapid assay test kit (SpotSense salivary CRP rapid test kit). This is a quantitative immunochromatographic test. It is a pre-commercial device that is currently being used for the evaluation of CRP. These kits were characterized and calibrated for newborn saliva [13].

**Figure 1:**
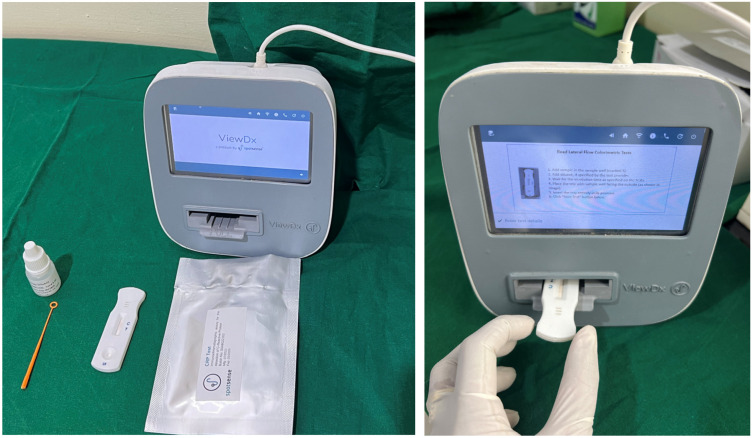
Armamentarium used for study: saliva collection kit (sample collection loop and buffer solution) and CRP test kit along with VIEWDx analyzer.

One drop of freshly collected unstimulated saliva (approximately 20 mL) was added to the test end of the kit, followed by the addition of two drops of the buffer solution, primarily composed of saline. This buffer solution ensured proper sample flow on the test card, aiding in uniform distribution for accurate results. After the addition of the sample and buffer solution, the kit was observed for 20 min for the appearance of the test line and control line. The test line’s presence indicated the presence of CRP in the saliva sample, while the control line served as a validation for test accuracy (Figure 1).

After 20 min, the test was read using a specialized readout device, providing precise measurements of the CRP levels in the saliva samples.

The salivary CRP levels were entered onto a Microsoft Excel spreadsheet (Microsoft Corp., Redmond, WA, USA) and were statistically analyzed by using SPSS software version 20 (IBM, Armonk, NY, USA). Kruskal–Wallis ANOVA with a post hoc Dunn’s test were applied to determine the difference in the mean and standard deviation values between the case and control groups. Area under ROC analysis was performed to identify the sensitivity and specificity, and the positive and negative likelihood ratios of the salivary CRP values in ruling out the disease. Multinomial regression was used to evaluate the role of the predictor (salivary CRP levels) on the outcome (cancer/OPMDs).

## 3. Results

The overall sample size was 109 subjects. Table 1 shows the age and gender-wise distribution of the study samples. The mean age was 53.50 years for the oral cancer group, 44.08 for the OPMD group and 50.33 for the normal healthy controls. The study sample included 29 biopsy-proven cases of oral squamous cell carcinoma, 40 biopsy-proven cases of OPMDs and 40 healthy controls (Table 2). Most of the cases of oral cancer were seen in the buccal mucosa (13), alveolar ridge (8), tongue (7) and alveolus along with the maxillary sinus (1).

Amongst the 29 patients in the oral cancer group, 2 patients had OSMF with malignant transformation (clinical staging of T3N0Mx and T3N0M0 and histopathological staging of T4aN2bM0 and T4aN0M0). Four patients were clinically diagnosed with leukoplakia, but histopathological diagnosis revealed squamous cell carcinoma; hence, they were shifted to the malignancy group. The remaining 23 patients had oral squamous cell carcinoma at histopathological stage III (4 patients) and stage IV (19 patients).

The mean calculated salivary CRP level detected in the oral cancer group was 4.21 ng/mL (SD = 7.05, median = 1.20, interquartile range = 0.36–4.19). The mean calculated salivary CRP level detected in the OPMD group was 2.51 ng/mL (SD = 3.19, median = 0.68, interquartile range = 0.41–5.99) and was 0.7 ng/mL (SD = 1.20, median = 0.31, interquartile range = 0.25–0.64) for the control group (Table 2 and Table 3). An overall significant difference in the salivary CRP levels between the study groups was noted (*p* < 0.001).

Post hoc tests showed that the salivary CRP levels were significantly higher in the oral cancer and OPMD groups than in the controls (controls vs. OPMDs: *p* = 0.01, controls vs. oral cancer: *p* = 0.001 and OPMDs s. oral cancer: *p* ≥ 0.99). No significant differences were observed between the oral cancer and OPMD groups.

**Figure 2:**
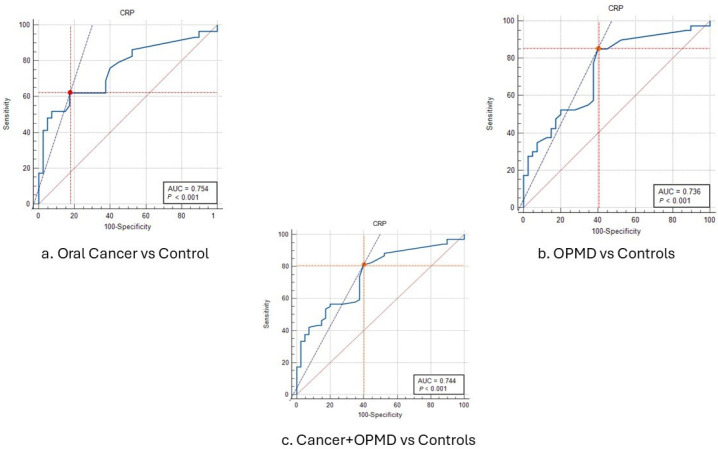
(**a**) The receiver operating characteristic curve for distinguishing patients with oral cancer from healthy controls based on salivary CRP values. (**b**) The receiver operating characteristic curve for distinguishing patients with OPMDs from healthy controls based on salivary CRP values. (**c**) The receiver operating characteristic curve for distinguishing patients with oral cancer and OPMDs combined from healthy controls based on salivary CRP values.

The sensitivity and specificity for oral cancer versus the control group were 62.07% and 82.5%, respectively, which resulted in positive and negative likelihood ratios of 3.55 and 0.46, respectively. Area under the ROC curve (AUC) was 0.75 and the Youden index was 0.45 (Table 4). However, the sensitivity and specificity for the OPMDs versus the control group were 85% and 60%, respectively, which resulted in positive and negative likelihood ratios of 2.12 and 0.25, respectively. The AUC was 0.74 and the Youden index was 0.45. When the oral cancer and OPMD groups were combined and compared against the control group, the sensitivity and specificity were 81.16% and 60%, respectively, which yielded a positive and negative likelihood ratio of 2.03 and 0.31, respectively. The AUC was 0.74 and the Youden index was 0.41. Figure 2 depicts the ROC characteristics comparing all three groups. 

As all the ROC curves showed, the AUC of above 0.7 implied good diagnostic accuracy. The overall positive likelihood ratios were lower; this implied that this cut-off level of salivary CRP cannot be considered for confirming the disease. However, based on the negative likelihood ratios, the above chosen cut-off levels of salivary CRP may be used to rule out the disease.

Multinomial regression was used to evaluate the role of the predictor (salivary CRP levels) on the outcome (cancer/OPMDs) adjusting for sex and age (Table 5). The regression analysis showed that compared to the control group, cancer (OR: 1.77) and OPMDs (OR: 1.67) had a significant association with salivary CRP levels after adjusting for age and sex. Owing to the high predilection of habit history, oral cancer and OPMDs in males, there was residual confounding for sex in the regression model.

## 4. Discussion

C-reactive protein is a homopolymeric protein synthesized by variety of cell types, such as hepatocytes, macrophages, smooth muscles, lymphocytes and endothelial cells. CRP binds to damaged cell membranes; hence, its serum level increases during severe inflammation and chronic inflammatory conditions, like malignancies and autoimmune systemic diseases. Inflammatory cells and cytokines influence tumor growth, tumor progression and immune suppression. Interleukin-1 and 6 are also linked to tumorigenesis. This suggests the existence of a systemic link between chronic inflammation and cancer. CRP is a validated inflammatory marker used frequently in clinical set-up for various autoimmune diseases and visceral malignancies [6].

The novel salivary CRP test kit used in our study is a bedside, colorimetric, lateral flow assay for the quantitative estimation of C-reactive protein in whole saliva. It measures C-reactive protein levels in systemic and oral inflammation in newborns and adults. The foremost study conducted using this kit was to predict culture-positive sepsis in neonates in the saliva and serum of neonatal patients [13]. The results were promising in predicting sepsis, and the salivary CRP cut-off scores were comparable to with serum CRP levels [13]. The present study is the second research study using this novel kit and the first-ever in the literature to detect chair-side salivary CRP in OPMD and malignancy patients. The objective of our study was to confirm the efficacy of a colorimetric, quantitative rapid assay kit in detecting and differentiating the salivary C-reactive protein in whole saliva samples of patients diagnosed with OPMDs and oral malignancy and differentiate it from normal saliva based on the CRP values. The results of the study showed significantly higher values for salivary CRP levels in the OPMD (mean = 2.51 ng/mL) and oral cancer groups, compared to the healthy controls. The oral cancer patients showed the highest level of CRP, with a mean value of 4.21 ng/mL.

A study by Kaur M U et al., showed a mean value of 7.31 ± 3.34 mg/L salivary CRP in leukoplakia patients and a mean value of 5.92 ± 2.76 mg/L in OSMF patients [10]. A study by Gosavi and Torkadi compared the serum CRP levels of 150 study participants including 50 with oral submucous fibrosis, 50 with oral squamous cell carcinoma and 50 normal controls. They observed a mean serum value of 5.40 mg/L in the OSMF patients and 12.17 mg/L in the squamous cell carcinoma patients, respectively [14]. The results of these studies were similar to our observation, as they showed a trend of a significant increase in salivary CRP values in OPMD and malignancy patients compared to healthy controls, similarly to our study [10,15] However, the CRP values recorded in our study could not be correlated directly with the previous studies’ results due to the different laboratory techniques and working principle employed. Jablonska et al., in their study, stated a significant correlation between serum CRP levels and clinical stages of OSCC [15,16]. Patients with more advanced tumor stages also showed elevated levels of CRP in their serum, and these increased levels may indicate poor prognosis and tumor progression [17]. However, further longitudinal studies with larger samples are warranted to prove the method’s efficacy in the early detection of malignant transformation.

A rapid salivary test kit is necessary, as surveys indicate that patients are more likely to accept a non-invasive screening test over an invasive prick test [12]. CRP has been used as a diagnostic marker for various visceral organ malignancies [18]. Its use as a marker of oral malignancy in salivary samples has been evaluated by many researchers and reviewers including Brailo et al., 2006, Katakura et al., 2015 and Sato et al., 2010 [17,18,19]. However, this is the first study in the literature to use a rapid chair-side test kit for salivary CRP detection. The results of our study demonstrated a diagnostic cut-off value of >0.67 ng/mL of salivary CRP for differentiating cases (OPMDs + cancer) from the normal controls. Similarly, a cut-off value of >0.64 ng/mL was obtained to differentiate between oral cancer and the controls. Although previous studies have reported higher salivary CRP values in OPMDs and oral cancer, none of them has stated a diagnostic cut-off value. Our study showed a sensitivity of 81.16%, which is a promising result for employing this kit as a chair-side screening tool for differentiating normal samples from OPMDs and oral cancer.

The few limitations of our study are that a comparison between serum and salivary CRP values could not be included as serum tests are invasive and have less patient compliance. The sample distribution in the study was unequal, with a higher number of males than females as a higher number of males engage in the habits of gutka, arecanut and tobacco consumption in our region. To strengthen the findings, future research should aim for equal sample sizes for both genders. This would help reduce bias and improve the generalizability of the results. We considered histopathology as the gold standard for definitive diagnosis of these lesions. However, a long-term study with a higher sample size and comparison between serum and salivary CRP levels could further unfold the efficacy of this test kit as an early diagnostic tool.

## 5. Conclusions

The present study showed that the novel rapid salivary test kit could significantly differentiate between the salivary CRP values of healthy controls and those of OPMD and malignancy patients. However, there was no significant difference in the CRP values between the OPMD and malignancy groups. It also demonstrated higher sensitivity values, confirming the efficacy of this kit as a screening tool. Further long-term studies with larger sample size are needed to assess if a biological cut-off value of salivary CRP can differentiate the OPMD group from the malignancy group. Also, diverse studies could be planned to assess salivary CRP diurnal variations, variations based on tumor staging and the post-treatment prediction of prognosis. This novel kit may also offer benefits as an objective educational tool for tobacco users who are at risk of developing OPMDs and oral cancer.

## Figures and Tables

**Table 1:** Age distribution of study subjects.

	Groups					
	Oral Cancer (n = 29)		OPMDs (n = 40)		Controls (n = 40)	
	Mean	SD	Mean	SD	Mean	SD
**Age**	53.50	12.49	44.08	12.18	50.33	12.96
**Gender-wise distribution**	**N**	**%**	**N**	**%**	**N**	**%**
Female	3	10.3%	7	17.5%	19	47.5%
Male	26	89.7%	33	82.5%	21	52.5%

**Table 2:** The median and quartile-wise distribution of the salivary CRP values in the sample data.

	Mean (Salivary CRP in ng/mL)	SD	Median	Percentile 25	Percentile 75
Oral Cancer	4.21	7.05	1.20	0.36	4.19
OPMDs	2.51	3.19	0.68	0.41	5.99
Controls	0.70	1.20	0.31	0.25	0.64

**Table 3:** The mean salivary CRP levels in all three groups.

	Group									*p*-Value	Post Hoc Test
	Oral Cancer			OPMDs			Controls				
	Mean	SD	N	Mean	SD	N	Mean	SD	N		
Salivary CRP (ng/mL)	4.21	7.05	29	2.51	3.19	40	0.7	1.2	40	<0.001; Sig	Oral Cancer, OPMD > Controls

**Table 4:** Summary of receiver operating characteristic curves for salivary CRP levels for oral cancer or OPMDs or both oral cancer and OPMD conditions in comparison with healthy controls.

	Global Measures		Sensitivity	Specificity	PPV	NPV	Cut-Off CRP	+LR	−LR
	AUC (95% CI)	Youden Index							
**Oral Cancer**	0.75 (0.64–0.85)	0.45	62.07	82.5	72%	75%	>0.69	3.55	0.46
**OPMDs**	0.74 (0.63–0.83)	0.45	85	60	68%	80%	>0.33	2.12	0.25
**Oral Cancer + OPMDs**	0.74 (0.65–0.82)	0.41	81.16	60	77.78%	64.86%	>0.33	2.03	0.31

PPV: Positive predictive value; NPV: Negative predictive value; AUC: Area under the Curve; CI: Confidence Interval; +LR: Positive likelihood ratio; −LR: Negative likelihood ratio; OPMDs: Potential Malignant Disorders.

**Table 5:** Multinomial regression to evaluate the role of predictor (salivary CRP levels) on the outcome (cancer/OPMDs), adjusting for sex and age.

Group		*p*-Value	OR (95% CI)
Cancer	Intercept	0.003	
	CRP	0.001	1.77 (1.25–2.5)
	Age	0.253	1.03 (0.98–1.08)
	Male	0.001	14.78 (2.89–75.57)
OPMDs	Intercept	0.986	
	CRP	0.003	1.67 (1.19–2.36)
	Age	0.068	0.96 (0.92–1)
	Male	0.007	5.19 (1.55–17.31)

## Data Availability

The data that support this study are available from the corresponding author upon reasonable request.
